# Hepatobiliary cancers digital twins: Integrating artificial intelligence-driven multi-omics with organoid dynamics

**DOI:** 10.1515/jtim-2026-0060

**Published:** 2026-10-23

**Authors:** Kun Liu, Fan Yang, Xuanying Lyu, Huisi He, Wen Wen

**Affiliations:** National Center for Liver Cancer, Third Affiliated Hospital of Naval Medical University (Second Military MedicalUniversity), Shanghai, China; Department of Laboratory Diagnosis, Third Affiliated Hospital of Naval Medical University (Second MilitaryMedical University), Shanghai, China; Department of Oncology, Third Affiliated Hospital of Naval Medical University (Second Military MedicalUniversity), Shanghai, China

**Keywords:** hepatobiliary cancers, digital twin, patient-derived organoids, spatial multi-omics, artificial intelligence

## Abstract

Static biomarkers are increasingly failing to predict outcomes in hepatobiliary cancers, largely due to the profound heterogeneity of the tumor microenvironment. Over the past decade, rapid advances in digital pathology, single-cell profiling, and spatial multi-omics have generated increasingly detailed maps of tumor architecture. Yet, these static snapshots fail to capture the dynamic cellular interactions driving disease progression. Conversely, patient-derived organoids (PDOs) and organ-on-chip models capture functional drug responses but often lack the microenvironmental complexity of the original tumor. In this review, we use the term digital twin to refer to a patient-specific model that is initialized with individual baseline data, linked to measurable biological or clinical readouts, and refined with longitudinal observations. We outline a unified three-layer architecture: A Data Layer (digitizing static atlases), a Physical Layer (engineering high-fidelity PDO avatars using tunable biomaterials), and a Computational Layer (integrating PDOs and patient data). Moreover, we discuss clinical applications, including individualized therapy selection, exploration of resistance mechanisms, and optimization of drug combinations and treatment sequences. Finally, we highlight the major barriers to clinical translation, including limited paired datasets, variable PDO feasibility, and the difficulty of integrating data collected at different time scales. Taken together, these advances may support more individualized and adaptive care in hepatobiliary cancers.

## Introduction

Hepatobiliary cancers comprise three primary entities: Hepatocellular carcinoma (HCC), cholangiocarcinoma (CCA), and gallbladder cancer (GBC), with CCA and GBC commonly grouped as biliary tract cancers (BTCs).^[1]^ Despite advances in diagnosis and treatment, these tumors remain highly lethal, largely owing to pronounced inter- and intratumoral heterogeneity shaped by their distinct tissue contexts. HCC typically arises within vascular-rich lesions embedded in a relatively soft hepatic parenchyma, whereas BTC lesions frequently develop within densely fibrotic, poorly vascularized stroma that imposes a physical barrier to drug penetration and immune-cell infiltration.^[2,3]^ The dominant microenvironmental features, therefore, differ between subtypes: In HCC, chronic inflammation and cirrhosis shape complex immune-infiltration patterns, whereas in BTC, profibrotic reactions and dense stromal barriers are the primary determinants of therapeutic resistance.^[4,5]^ Current stratification systems based on staging, etiology, anatomic location, and genomics capture only fragments of this complexity and insufficiently reflect microenvironmental constraints, contributing to heterogeneous and often suboptimal therapeutic responses.^[6, 7, 8]^

Recent progress in spatial transcriptomics, multiplexed imaging, and single-cell multi-omics has enabled increasingly detailed maps of hepatobiliary tumor ecosystems, revealing spatially organized niches of immune evasion, stromal remodeling, and therapeutic resistance.^[2]^ While spatial transcriptomics has generated exquisitely detailed maps of the tumor ecosystem, a critical limitation remains: These maps are frozen in time. In parallel, patient-derived organoids (PDOs) and other three-dimensional culture systems have emerged as platforms that retain key genomic and phenotypic features of primary tumors, providing experimental access to clonal dynamics, drug responses, and patient-specific matrix cues that are central to BTC desmoplasia and HCC vascularity.^[9,10]^ Together, these “static” and “dynamic” modalities point to a practical need: A framework that can integrate multiscale, multimodal data to approximate the evolving behavior of hepatobiliary cancers.

Building on these advances, the concept of a medical digital twin has emerged as a framework for integrating heterogeneous data streams into patient-specific, evolving models that can support prognosis, risk assessment, and treatment planning.^[11]^ Here, we use an operational definition of a digital twin: A patient-specific computational representation that is explicitly linked to its physical counterpart and can be iteratively updated as new experimental or clinical observations become available.^[12]^ Under this definition, a digital twin extends beyond static prediction by remaining linked to longitudinal measurements and allowing recalibration when predicted and observed behaviors diverge.^[13]^ This narrower use of the term is intentional because recent reviews have shown substantial heterogeneity in how patient digital twins are defined and implemented in clinical research.^[14]^

Within hepatobiliary cancers, a digital twin must integrate tissue-scale atlases, functional readouts from PDOs, and longitudinal clinical information into a representation that can be refined as new data accumulate.^[12]^ Achieving this integration depends on connecting static spatial and molecular profiles with dynamic measurements obtained from biomaterial-engineered PDO systems, alongside imaging and routine clinical data, to approximate how individual tumors evolve.^[15,16]^ In this review, we focus on a near-term, translational form of digital twins that can be constructed and assessed using current experimental and clinical resources, rather than on fully autonomous or real-time decision systems.^[12,17,18]^ Based on this scope, we introduce a three-layer framework consisting of a Data Layer, a Physical Layer, and a Computational Layer, which provides a structured path from descriptive mapping to predictive modeling of treatment response (Figure 1). A minimum viable product (MVP) workflow, together with indicative timelines, is summarized in Table 1.

**Figure 1:**
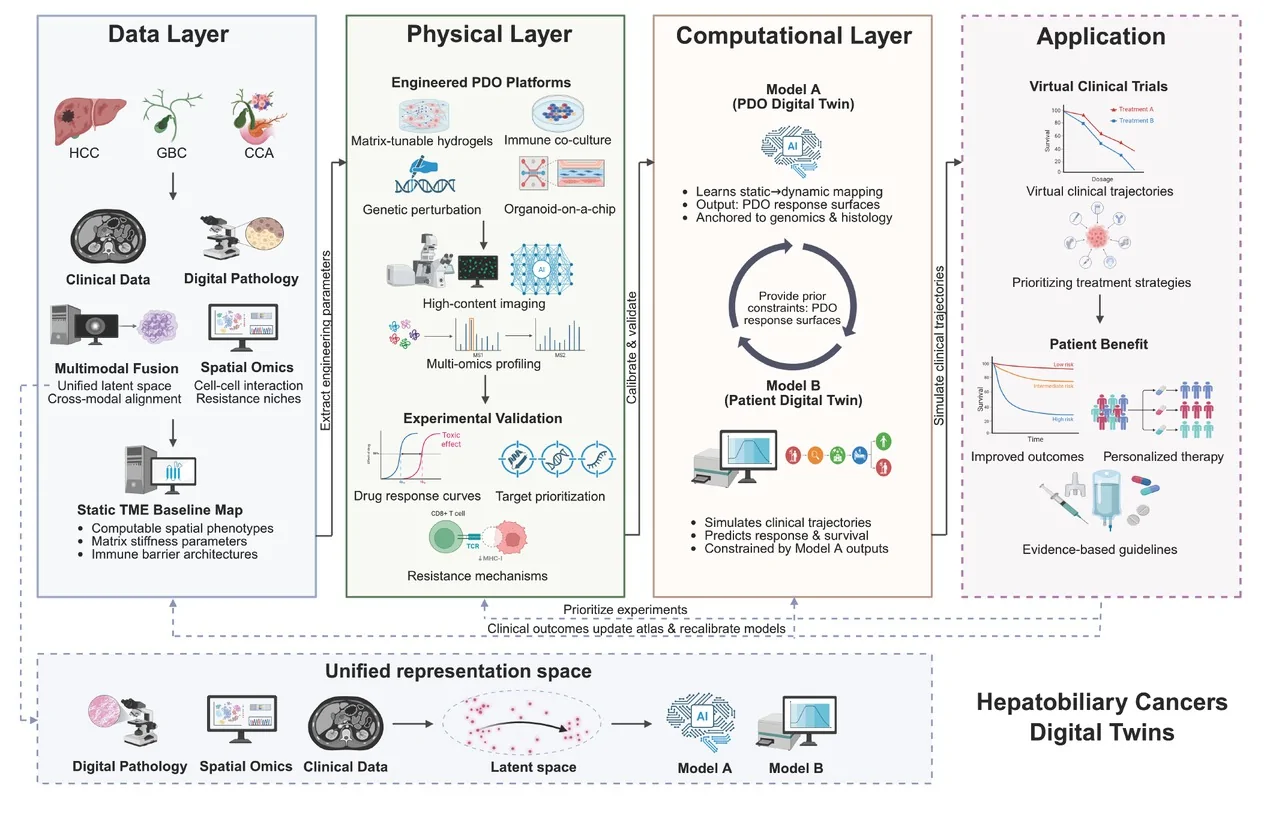
Hepatobiliary cancers digital twin system architecture. This diagram outlines a multi-layer framework integrating heterogeneous data and models to support precision oncology. The Data Layer transforms clinical data, digital pathology, and spatial omics into a computable static TME baseline. The Physical Layer employs engineered PDO platforms, incorporating matrix-tunable hydrogels, immune co-culture, HCI, and multi-omics profiling for experimental validation of drug responses and resistance mechanisms. The Computational Layer hosts Model A (which learns how atlas-derived baseline states relate to PDO phenotypes) with Model B (which simulates patient-level clinical trajectories constrained by Model A). The Application section enables virtual clinical trials, prioritizes treatment strategies, and links predictions to patient benefit. Clinical outcomes feedback to update the atlas and recalibrate the coupled models, thereby closing the loop. An inset illustrates how heterogeneous modalities are aligned into a unified representation space. This space defines the patient-specific baseline state used to constrain downstream computational modules (Model A and Model B). HCI, high-content imaging; TME, tumor microenvironment; PDO, patient-derived organoid; HCC, hepatocellular carcinoma; GBC, gallbladder cancer; CCA, cholangiocarcinoma. Created in BioRender (2026). (https://BioRender.com/ax3qsy0).

**Table 1:** MVP workflow for hepatobiliary cancers

Workflow stage	Typical sample source	Typical turnaround time (indicative)	Minimal inputs	Role in the Atlas-PDO-Twin integration	Fallback when PDO not feasible
Digital pathology baseline	FFPE biopsy or resection	1-3 days	H&E whole-slide images	Initializes spatial and morphological atlas state	Always available; anchors reduced twin configuration
Bulk molecular profiling (optional)	Fresh or FFPE tumor tissue	7-14 days	Targeted DNA panel or bulk RNA-seq	Refines molecular baseline and subtype priors	Omitted without blocking MVP
Spatial molecular profiling (optional)	Fresh-frozen tissue	2-4 weeks	Spatial transcriptomic or proteomic maps	Adds spatial constraints and niche-level resolution	Skipped in time-critical settings
PDO establishment	Fresh biopsy or surgical tissue	2-4 weeks	Viable tumor fragments	Provides dynamic ex vivo functional surrogate	Atlas-only twin when establishment fails
PDO perturbation assays	Established PDO cultures	7-14 days	Growth kinetics; treatment response phenotypes	Constrains functional response parameters	Early/partial readouts used if full assays exceed time window
Model initialization and updating	Integrated patient data streams	1-3 days	Atlas features ± PDO-derived constraints	Couples’ atlas state with functional and clinical evolution	Reduced-order model without PDO priors

This table summarizes a practical near-term workflow designed to operate within current experimental and clinical constraints. The turnaround times are indicative and combine reported experience from organoid-guided pipelines with practical assumptions where hepatobiliary-specific evidence remains limited. MVP: minimum viable product; PDO: patient-derived organoid.

At the same time, integration across data types cannot rely on a shared latent representation alone. Instead, it requires concrete points of alignment that link measurements across modalities and time. In this context, we describe practical anchors, including the use of samples from the same lesion or timepoint, registration between histology and spatial profiling on adjacent sections, and common histopathologic readouts such as immunohistochemistry, immune composition, or fibrosis-related features. We also outline a concise set of reporting items intended to support the evaluation of translational digital twin studies, while recognizing that such practices have not yet been standardized or validated for hepatobiliary cancer datasets (Table 2).

**Table 2:** Integration anchors and recommended reporting items for hepatobiliary cancer digital twins

Category	Item	Purpose	Practical limitations
Integration anchors	Same-lesion alignment	Ensures correspondence across modalities derived from the same tumor lesion	Limited by tissue availability
Integration anchors	Same-timepoint alignment	Reduces temporal drift between measurements	Constrains data completeness
Integration anchors	Adjacent-section registration	Enables alignment between WSI and spatial profiling from serial sections	Susceptible to sectioning artifacts
Integration anchors	Shared histopathologic observables	Provides interpretable weak anchors (e.g., IHC markers, immune composition, fibrosis surrogates)	Lower specificity than direct co-registration
Evaluation and reporting	External validation	Assesses generalizability across independent cohorts	Often limited by data access
Evaluation and reporting	Calibration	Evaluates agreement between predicted and observed outcomes over time	Distinct from discrimination metrics
Evaluation and reporting	Robustness to missing modalities	Tests performance under partial data scenarios	Essential for real-world deployment
Evaluation and reporting	Uncertainty reporting	Quantifies and communicates predictive uncertainty	Required for clinical interpretability
Evaluation and reporting	Transparency of integration	Explicitly documents how modalities are aligned and weighted	Avoids implicit latent-space assumptions

These integration anchors and reporting items are proposed as plausible and recommended practices for translational digital twin studies. Their inclusion does not imply that standardized or validated implementations currently exist for hepatobiliary cancer datasets. WSI: whole-slide image; IHC: immunohistochemistry.

### Box 1| operational definition of a medical digital twin

In this review, a medical digital twin is defined as a patient-specific computational model that: (1) is initialized using data from an individual patient; (2) can be iteratively updated as new experimental or clinical observations become available; (3) is calibrated against longitudinal measurements to maintain consistency between predicted and observed behaviors; (4) reports predictive uncertainty when possible, rather than relying on point estimates alone; (5) documents model inputs and updates clearly so that they can be reviewed and evaluated.

This operational definition is also consistent with recent cross-disciplinary guidance that emphasizes personalization, dynamic updating, predictive capacity, and decision support as core elements of a digital twin.^[14]^ Models that lack patient-specific linkage or iterative calibration—such as static prognostic artificial intelligence (AI) models or cohort-level in silico simulations—are considered complementary approaches but do not constitute digital twins under this definition.

## The data layer: digitizing the microenvironment landscape

In the digital twin architecture, the Data Layer transforms unstructured histology and high-dimensional omics into computable spatial phenotypes. These representations provide initial-state parameters for the twin and the physical design constraints for bioengineered models. In hepatobiliary cancers, this layer digitizes the tumor microenvironment (TME) by converting whole-slide images (WSIs) and spatial molecular readouts into quantitative descriptors of vascularity, stromal composition, and immune-tumor topology, thereby linking microscopic structure to model initialization and material design.^[4,19,20]^

### Extracting physical constraints from digital pathology

Among clinical data streams in hepatobiliary cancers, hematoxylin and eosin (H&E) WSIs are one of the most ubiquitous and historically accumulated resources in the field. AI enhances digital pathology through quantitative parsing of histological features, phenotypic reconstruction of microenvironmental patterns (*e.g*., immune-desert phenotypes), and cross-modal inference of molecular states.^[5,19]^ Hepatobiliary cancers exhibit marked heterogeneity in vascularity and stroma: HCC is typically vascular-rich with limited desmoplasia, whereas BTC is characterized by dense fibrotic stroma. Practically, the most informative histology-derived spatial features differ by entity. For HCC, histology-derived features related to vascular interfaces, such as tumor-endothelial adjacency and vascular invasion surrogates, may be more informative than collagen abundance alone.^[21,22]^ For BTC, the dominant signal often resides in the stroma, including collagen-rich belts, CAF density gradients, and ductal-stromal boundaries that shape immune exclusion and drug penetration.^[23,24]^ Consistent with this BTC-focused emphasis, quantitative analysis of collagen fiber density, orientation, and spatial continuity in CCA and GBC provides surrogates of matrix stiffness and barrier properties, and has been associated with prognosis as well as with the design space for hydrogel or organoid scaffolds.^[25]^

Once tissue structures and cell populations are segmented, WSIs can be represented as “cell graphs” that encode the spatial relationships between tumor, immune, and stromal cells. Graph- and topology-based features, such as immune-tumor distances, clustering of myeloid cells and fibroblasts, and the continuity of stromal belts at the tumorstroma interface, offer compact quantitative descriptors of immune-desert, immune-excluded, and inflamed architectures.^[26, 27, 28]^ These image-derived phenotypes bridge histological patterns with risk stratification and provide computable constraints for constructing microenvironment-informed dynamic models.

### Deciphering resistance niches using spatial omics

Within the digital twin framework, spatial omics provides an *in situ* molecular coordinate system in which cell types, states, and neighborhood relationships are measured while preserving tissue architecture.^[29,30]^ Compared with morphology alone, these data enable precise annotation of cell types and functional states, and allow reconstruction of spatial units such as ring-shaped immune barriers, immunosuppressive niches formed by myeloid cells and fibroblasts, and boundary structures at invasive fronts.^[26]^

In hepatobiliary cancers, spatial analyses have been used to dissect mechanisms of therapeutic resistance that arise from spatial organization rather than intrinsic tumor cell states. For example, in HCC, dense myeloid-fibroblast belts surrounding tumor nests limit effector T-cell penetration into tumor cores, and correlate with poor outcomes under immunotherapy^[26]^. Other studies that integrate tissue architecture with cellular composition in HCC and BTC have revealed regional variation in immune microenvironments and identified immune-desert, immune-excluded, and inflamed regions within the same lesion.^[27,28]^ In intrahepatic CCA (ICCA), spatial transcriptomics has highlighted tumor-associated macrophages (TAMs) and other key populations occupying central positions in intercellular communication networks, emphasizing the need to explicitly represent region-specific immunosuppressive structures in hepatobiliary digital twins.^[31,32]^ Collectively, these findings illustrate how spatial omics can move from static descriptive maps toward mechanistic explanations of immune escape and drug resistance shaped by spatial barriers.

This distinction matters for twin design. Notably, what constitutes a clinically relevant “barrier” is not the same in HCC and BTC. In HCC, perivascular niches and endothelial programs can dominate local immune states, so spatial features that resolve tumor-endothelial proximity and vascular-associated immune zoning are high-priority outputs.^[33,34]^ In iCCA and extrahepatic CCA (eCCA), immune exclusion is frequently structured by CAF-rich desmoplastic corridors and macrophage-fibroblast interaction hubs within the tumor stroma. As a result, stromal density maps and interaction modules centered on CAF and TAMs represent key spatial features that must be preserved during digital-twin initialization.^[23,31]^

Spatial omics remains difficult to standardize for routine clinical workflows. Practical bottlenecks include high assay cost, variable tissue handling requirements (especially for FFPE compatibility), and uneven turnaround times across platforms, which often do not match clinical decision windows.^[35,36]^ Data sparsity and technical noise can also propagate into cell-type calling and neighborhood inference, and segmentation errors in imaging-based assays can bias downstream spatial interaction analyses.^[37,38]^ Cross-site comparability is further limited by inconsistent gene panels and the absence of universally adopted performance metrics, although recent community efforts have started to define standardized evaluation datasets and reproducibility metrics.^[39,40]^ These constraints imply that near-term digital twins should treat spatial assays as optional, high-value inputs with explicit quality control (QC) and reporting, rather than as mandatory components for every patient.^[37]^

### Constructing a unified latent space through multimodal fusion

Digital pathology excels at capturing morphological patterns, whereas spatial omics provides an *in situ* molecular coordinate system. Yet neither modality alone can fully account for the complexity of the hepatobiliary TME. The central goal of multimodal integration is therefore to construct a unified representation space in which different data types can be aligned, cross-validated, and jointly used to predict clinical phenotypes and treatment responses.^[20,41]^ The lower inset of Figure 1 schematically illustrates how digital pathology, spatial omics, and clinical data are aligned into a unified representation space that supports downstream modeling. At the static mapping layer of a digital twin, such a representation encodes cellular composition, spatial structure, and molecular programs, while exposing interfaces for integration with PDO-based functional readouts and longitudinal clinical outcomes.

In the single-cell multimodal setting, joint modeling frameworks embed ribonucleic acid (RNA), chromatin accessibility, and protein measurements into a shared latent space, capturing discrete cell identities as well as continuous cell-state trajectories, and enabling cross-modal alignment and imputation.^[42]^ For histology-omics integration, combining deep representations of WSIs with genomic and transcriptomic profiles has improved molecular subtyping and prognostic modeling over single-modality approaches, and has linked visible morphology to genotype and pathway activity.^[43, 44, 45]^ Proteogenomic studies add a functional dimension by jointly interrogating proteomes and genomes, revealing subtypes more closely tied to pathway activity, druggable targets, and TME states.^[46,47]^ Complementary to tissue-based atlases, computed tomography (CT), magnetic resonance imaging (MRI), and functional imaging provide non-invasive, longitudinal views of hepatobiliary tumors. Radiomics pipelines that extract tumor shape, texture, enhancement dynamics, and vascular invasion patterns have shown value for staging, molecular surrogacy, and treatment-response prediction in HCC and BTC, and can serve as patient-level constraints and validation targets for digital-twin trajectories.^[48,49]^ For HCC, dynamic enhancement patterns and vascular invasion-related imaging features provide natural longitudinal constraints that complement tissue snapshots.^[22,50]^ For BTC, imaging and laboratory trajectories reflecting biliary obstruction (*e.g*., ductal dilatation and bilirubin dynamics) are often the rate-limiting constraints that determine whether systemic therapy can be delivered and therefore must be encoded explicitly.^[51]^ When these multimodal encoders are anchored as the Data Layer of a digital twin, they provide a computable, patient-specific baseline from which dynamic PDO responses and clinical trajectories can be simulated (Table 3). Recent ICC studies also suggest that pathomics-derived baseline features can serve as biologically grounded constraints for multimodal prediction models and may complement radiologic response assessment in longitudinal settings.^[53]^

**Table 3:** Multimodal data inputs for initializing a hepatobiliary cancer digital twin

Data modality	Primary data source	Key processing	Feature representations	Role in Data Layer	References
Digital pathology (H&E, multiplex IHC/IF)	WSIs; TMAs from tumors/biopsies	Stain normalization; artefact QC; tissue/cell segmentation; cell-graph construction	Cell-type density maps; spatial graphs; tumor-stroma interface metrics; MVI surrogates	Defines tissue-scale architecture and physical constraints (stroma, vascularity) for atlas initialization	[5,18,20,21]
Spatial omics (transcriptomics / proteomics)	Spatial transcriptomics; *in situ* RNA/protein profiling; multiplex protein imaging	Coordinate registration; batch correction; deconvolution; WSI alignment	Spatial programs/modules; ligand-receptor maps; niche/state annotations	Identifies spatial resistance niches and immune-stromal barriers for baseline state	[24, 25, 26, 27]
Bulk genomics & transcriptomics	WES/targeted sequencing; bulk RNA-seq	Variant/CNV calling; expression normalization; pathway scoring	Driver events; mutational signatures; pathway activity profiles	Supports molecular subtyping and anchors each case in atlas-defined baseline coordinates	[30, 31, 32]
Single-cell / multi-omic profiling	scRNA-seq/snRNA-seq; multi-ome assays	QC & doublet filtering; batch correction; cross-modal integration; annotation	Cell states; lineage/trajectory features; EMT/plasticity programs; chromatin accessibility states	Resolves cellular heterogeneity and supplies state priors for plasticity/resistance modeling	[29]
Proteogenomics / metabolomics	Global/phosphoproteomics; metabolomics/lipidomics	Identification/quantification; normalization; pathway aggregation	Kinase activity; signaling rewiring; metabolic signatures	Adds functional pathway constraints beyond genotype and informs mechanism-level parameters	[33,34]
Radiology & radiomics	Longitudinal CT/MRI (± functional imaging)	Lesion segmentation; intensity normalization; scanner harmonization	Shape/texture features; enhancement dynamics; invasion-related imaging signatures	Provides non-invasive longitudinal constraints for trajectory tracking and output validation	[35,36]
Clinical & laboratory variables	EHR; labs; treatment exposures; adverse-event reports	Unit harmonization; outlier handling; missingness modeling	Liver function panels; AFP/CA19-9; exposure curves; risk scores	Defines key covariates and clinical endpoints for calibration/validation	[10,52]
Multimodal latent representations	Integrated pathology + omics + imaging + clinical	Cross-modal registration; representation learning; harmonization	Shared embeddings; similarity maps; latent state trajectories	Builds a unified baseline representation to link modalities and support patient-specific initialization	[19,28]
PDO baseline omics & imaging	Baseline PDO sequencing & imaging (pre-perturbation)	Tumor-PDO alignment; PDO image feature extraction	PDO subtype signatures; baseline morphology metrics; pathway states	Aligns PDO avatars to atlas baseline states and sets starting conditions for perturbations	[37,38]

AFP: alpha-fetoprotein; CA19-9: carbohydrate antigen 19-9; CNV: copy number variation; CT: computed tomography; EHR: electronic health record; EMT: epithelial-mesenchymal transition; H&E: hematoxylin and eosin; IF: immunofluorescence; IHC: immunohistochemistry; MRI: magnetic resonance imaging; MVI: microvascular invasion; PDO: patient-derived organoid; QC: quality control; scRNA-seq: single-cell RNA sequencing; snRNA-seq: single-nucleus RNA sequencing; TMA: tissue microarray; WES: whole-exome sequencing; WSI: whole-slide image.

Collectively, AI-enabled fusion of digital pathology, spatial omics, and other molecular layers enables the construction of a high-dimensional, computable static TME baseline map for each patient that captures cellular composition, spatial organization, and functional molecular information.^[20,41]^ However, this baseline primarily reflects pre-treatment or single time-point states and lacks dynamic feedback on TME remodeling or clonal evolution under therapeutic pressure. Bridging this gap will require translating static features—particularly matrix stiffness and immune barrier architectures—into engineering design parameters and incorporating advanced biomaterials and PDO systems to reconstruct controllable *in vitro* models for drug and immune perturbations, thereby providing experimentally testable anchors for hepatobiliary digital twins.

## The physical layer: engineering high-fidelity dynamic avatars

As illustrated in Figure 2, quantitative features derived from digital pathology and spatial atlases can be translated into engineering constraints to construct patient-matched physical avatars for hepatobiliary cancers. Hepatobiliary cancers span a broad spectrum—from vascular-rich HCC to the highly desmoplastic microenvironments of ICC and GBC—resulting in substantial heterogeneity in stromal architecture, vascular supply, and immune organization. Digital pathology and spatial multi-omics can now describe these ecosystems at high resolution, but they largely remain snapshots and therefore offer limited insight into how tumor-microenvironment interactions shift under treatment. Critically, to translate the digital and physical realms, AI algorithms must first translate atlas features into engineering constraints. For instance, quantitative collagen signatures from pathology slides are converted into stiffness parameters (kPa) for hydrogel synthesis, ensuring the physical avatar replicates the specific desmoplastic barrier of the patient’s original tumor. Conversely, for HCC, atlas-derived vascular density and perivascular immune zoning can be translated into perfusion and oxygen-gradient settings in organoid-on-chip designs, aligning physical stimulation with the vascular-rich tumor context. In a digital-twin context, PDOs provide a practical dynamic counterpart: they retain key genetic and phenotypic features of the source tumor while supporting controlled perturbation and longitudinal measurement, allowing treatment responses and resistance evolution to be studied in a way that is both experimentally tractable and computationally integrable.^[54, 55, 56]^

**Figure 2:**
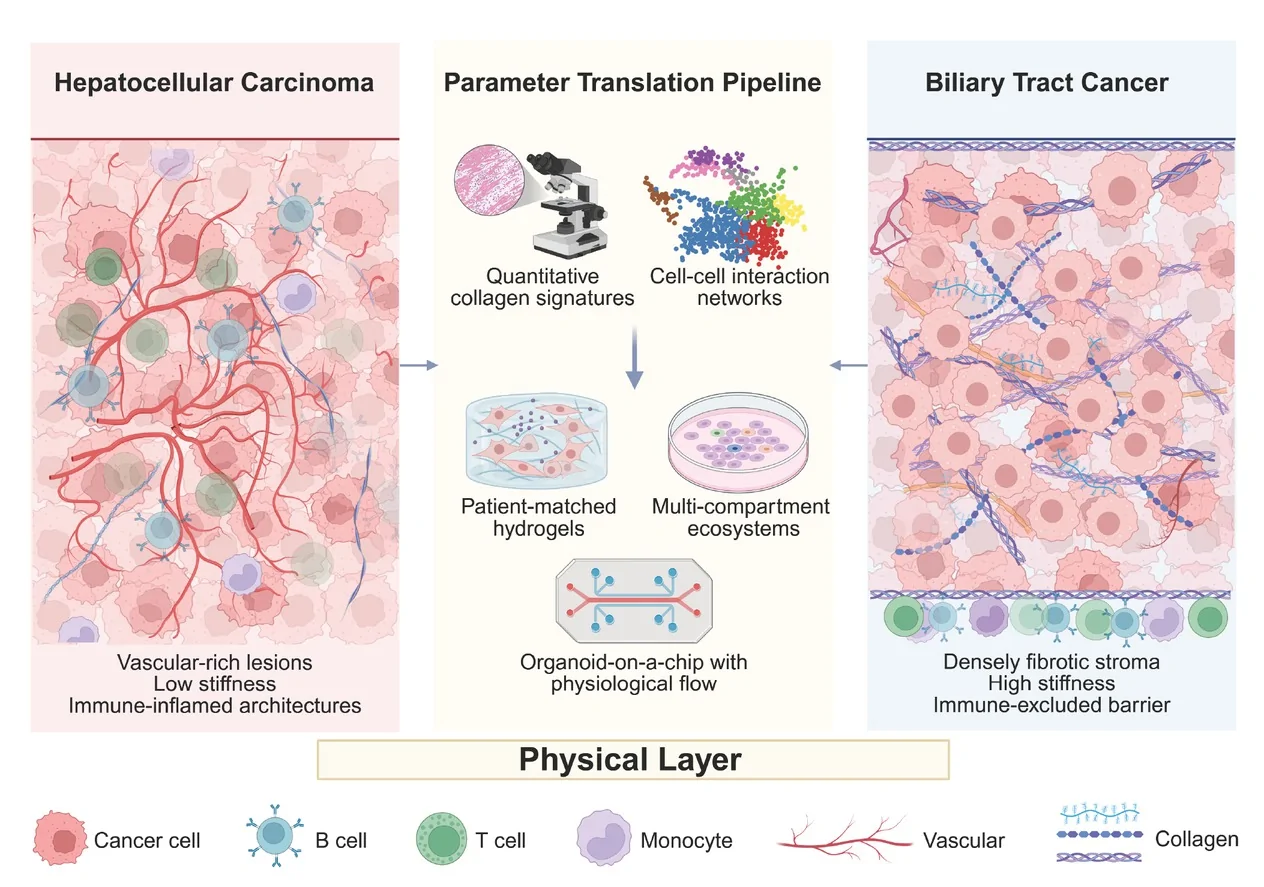
Engineering patient-matched physical avatars in hepatobiliary cancers. This schematic depicts the Physical Layer of a hepatobiliary cancer digital twin. A parameter-translation pipeline converts quantitative digital pathology features (e.g., collagen signatures, cell-cell interaction networks) into engineering constraints for patient‑matched hydrogel synthesis and multi‑compartment ecosystem construction. For HCC, vascular-rich lesions with low stiffness and immune‑inflamed architectures are modeled using organoid-on-a-chip systems with physiological flow. For BTC, densely fibrotic stroma with high stiffness and immune‑excluded barriers are replicated. HCC, hepatocellular carcinoma; BTC, biliary tract cancer. Created in BioRender (2026). (https://BioRender.com/n7juqfy).

### Establishment and microenvironmental reconstruction of hepatobiliary PDOs

Over the past decade, large PDO biobanks and systematic screening efforts have demonstrated that PDOs can capture substantial inter-patient variability in therapeutic responses and prospectively inform clinical decision-making across cancer types.^[10,54,57]^ In liver and BTCs, hepatobiliary PDO workflows have increasingly been coupled with multi-omics profiling and clinically annotated follow-up, supporting the idea that pharmacogenomic response patterns observed in patients can be reproduced and stratified in PDO-based models.^[58, 59, 60, 61]^ These experiences suggest that well-annotated hepatobiliary PDO pipelines can serve as a functional extension of molecular atlases rather than as isolated *in vitro* systems.^[55]^

A central challenge for hepatobiliary PDOs lies in reliably capturing the architectural and ecological complexity of the native tumors. In ICC and other BTCs, dense desmoplasia, vascular remodeling, and an immune-modulated milieu are difficult to reconstruct in standard cultures, which can bias clonal representation and distort drug-response readouts.^[62, 63, 64]^ The quality of hepatobiliary PDOs dictates the validity of downstream dynamic analyses. Systematic biases in clonal representation or drug response fidelity cannot be corrected by AI; algorithms will merely overfit these artifacts. If these biases are not controlled at the experimental level, downstream modeling will inherit them rather than resolve them.

To better approximate liver- and bile-duct-specific niches, recent work has focused on engineering extracellular matrices and stromal compartments. Synthetic or tissue-derived hydrogels with tunable stiffness, viscoelasticity, and ligand composition have underscored how matrix mechanics and biochemistry shape organoid morphology, lineage balance, and drug response beyond what conventional Matrigel can provide.^[62,63,65]^ In parallel, organoid-on-a-chip systems can impose controlled gradients of oxygen, nutrients, and soluble cues, enabling more stable long-term culture and higher-content functional readouts under physiologically relevant flow conditions.^[54,66,67]^

These engineering priorities also differ between hepatobiliary entities. In HCC, where tumors arise in a cirrhotic and highly vascularized organ, adding perfusion and oxygen gradients can be particularly informative for reproducing spatial heterogeneity that influences invasion and therapy response.^[68]^ In contrast, BTC—especially iCCA—is often shaped by a stiff, desmoplastic stroma; stiffness-tunable matrices and fibroblast-enriched cultures can better capture stromal control of invasion and stiffness-associated chemoresistance.^[52,69]^ These entity-specific constraints also help determine which stromal and immune compartments should be prioritized when moving from epithelial organoids toward multi-compartment ecosystems.

Beyond the extracellular matrix (ECM) tuning, hepatobiliary PDO platforms are increasingly moving toward multi-compartment ecosystems. Co-culture with cancer-associated fibroblasts, hepatic stellate cells, endothelial cells, and other stromal populations can partially reconstitute fibrotic and vascular compartments, and has been used to study how stromal remodeling modulates tumor growth and therapy sensitivity in liver cancer organoids.^[55,70,71]^ Immune-competent models, including CCA PDOs co-cultured with peripheral or tumor-infiltrating lymphocytes, macrophages, or natural killer (NK) cells, sometimes supported by microfluidic control of cytokine gradients, provide a tractable platform to examine immune engagement, cytotoxicity, exhaustion, and escape in a patient-specific manner.^[66,52,72]^ Together, these engineering strategies are gradually shifting hepatobiliary PDOs from simple epithelial cultures toward controllable ecological models with readouts that can be aligned to atlas features and clinical endpoints.

Engineering more “ecological” hepatobiliary PDO systems inevitably trades off against throughput, reproducibility, and cost. Adding stromal or immune compartments and moving to microfluidic organoids-on-chip introduces additional sources of variability (cell sourcing, ratios, activation states, perfusion settings), making cross-batch and cross-lab reproducibility harder without strict SOPs.^[73,74]^ Matrix choice remains a major confounder: Matrigel is convenient but poorly defined and batch variable, while synthetic or tissue-derived alternatives improve control yet are not universally standardized or scalable.^[75,76]^ Clinically, establishment success rates and timelines are heterogeneous in real-world pipelines, which can limit feasibility when treatment decisions are time-critical.^[77]^ Therefore, the review’s “near-term” digital twin should explicitly define a minimal viable PDO configuration and QC gates and treat higher-complexity co-cultures/chips as optional upgrades when time and resources permit.^[77]^

### High-content imaging (HCI) and AI for systematic phenotyping of tumor organoids

Because hepatobiliary PDOs are three-dimensional and morphologically diverse, bulk viability assays capture only a small fraction of their behavior. HCI enables time-resolved, volumetric measurement of organoid growth, death, invasion, and interaction with stromal or immune cells across hundreds to thousands of microcultures in parallel. For hepatobiliary cancers, this is particularly important for quantifying lumen formation, bile duct-like branching, matrix remodeling, and immune cell infiltration under treatment.^[78, 79, 80]^

Recent platforms combine microfabricated culture devices with fast light-sheet or confocal imaging and automated analysis to phenotype individual organoids at scale, including size, shape, internal architecture, and neighborhood context.^[78, 79, 80]^ Deep-learning pipelines such as Organoid further support semantic segmentation and tracking, enabling robust quantification of dynamic behaviors such as proliferation, budding, invasion, and collapse in response to perturbations.^[80,81]^

When coupled to systematic drug perturbations, HCI can describe a drug-induced morphological response space that complements conventional viability metrics by separating cytostatic from cytotoxic effects, capturing adaptive remodeling, and flagging tolerant subpopulations.^[82,83]^ Label-free imaging combined with machine-learning classifiers is particularly attractive for longitudinal monitoring of CCA PDO responses without invasive labeling.^[83]^ In immuno-oncology settings, behavior-resolved imaging platforms (*e.g*., BEHAV3D) and behavior-guided transcriptomics link T-cell migration, engagement, and killing dynamics to transcriptional states, offering a useful blueprint for extending similar readouts to hepatobiliary PDO-immune co-culture systems.^[84,85]^

Overall, HCI plus AI-based analysis transforms PDO assays from low-dimensional drug screens into rich phenotypic datasets that capture spatial and temporal patterns of treatment response. Within hepatobiliary digital-twin workflows, these high-dimensional phenotypes form a practical intermediate layer that can be aligned with pathology and multi-omics baselines and later integrated with transcriptomic, epigenetic, and metabolic readouts.^[80,82]^

### Multi-omics profiling: from phenotypic responses to resistance mechanisms

Multi-omics profiling adds a mechanistic layer to PDO phenotyping by linking response patterns to transcriptional, epigenetic, and metabolic programs. In hepatobiliary tumor organoids, pharmacogenomic and pharmaco-proteogenomic studies indicate that response heterogeneity reflects clonal architecture, differentiation state, and pathway wiring, and that integrating genomic, transcriptomic, and proteomic layers markedly improves drug-sensitivity prediction compared with single-modality readouts.^[58,59,86]^ More broadly, integrative multi-omics strategies developed in other tumor types provide a useful methodological foundation for dissecting intra-tumor heterogeneity in hepatobiliary organoid cohorts.^[59,60]^

Longitudinal single-cell and spatial multi-omics before and after treatment can further reconstruct state transitions associated with drug tolerance and acquired resistance. For example, drug-tolerant persister (DTP) states marked by stress-adaptive RNA-binding proteins such as Mex3a, together with chromatin remodeling programs, have been observed to survive cytotoxic pressure and may later seed resistant clones.^[87]^ Trajectory-inference and fate-mapping methods, including CellRank and related multi-omics integration pipelines, can place such intermediates along continuous state gradients and link signaling modules to distinct evolutionary routes.^[88,89]^

Metabolic rewiring is another determinant of organoid response. Spatial dynamic metabolomics and single-cell spatial metabolomics reveal how metabolic niches, nutrient competition, and oncometabolite accumulation influence cell fate decisions and drug sensitivity, providing a rationale for extending similar measurements to hepatobiliary PDOs and co-culture systems.^[90,91]^ Integrating metabolic maps with genomic and transcriptomic layers may help uncover lineage-specific liabilities not evident from expression data alone.^[90,91]^

Finally, organoid multi-omics datasets increasingly rely on computational approaches that embed heterogeneous modalities while retaining dynamic structure. Recent tools for multi-omics single-cell data integration and directed fate mapping support the construction of shared latent spaces that link baseline organoid states, DTP intermediates, and terminal resistant phenotypes, while genetic perturbation platforms such as in-organoid clustered regularly interspaced short palindromic repeats (CRISPR) screening and CRISPR-StAR enable systematic testing of inferred dependencies directly in three-dimensional cultures.^[92,93]^ In hepatobiliary cancers, the value of PDO multi-omics lies in linking high-content phenotypes to molecular trajectories and turning resistance hypotheses into experimentally testable modules that can later be incorporated into patient-level digital-twin frameworks.^[58,59,86]^ Together with the ECM engineering, organoid-on-a-chip, stromal co-culture, and immune-competent PDO platforms described above, these technologies form the core physical avatars and readouts of the Physical Layer (Table 4).

**Table 4:** PDO-based physical avatars and perturbation readouts that constrain a hepatobiliary cancer digital twin

Platform	Microenvironment configuration	Typical Perturbations	Key readouts	Role in Physical Layer	References
Baseline hepatobiliary PDO biobanks	ICC/ECC/GBC/HCC PDOs in standard ECM domes/scaffolds	SOC chemotherapy; targeted/kinase inhibitors; hypoxia/ischemia mimetics	Growth/viability; dose-response; morphology; lineage markers	Atlas-state-anchored *ex vivo* drug response; primary training/validation data for Model A	[16,17,40]
Engineered ECM and hydrogels	Defined hydrogels/scaffolds with tunable stiffness and ligands	Stiffness/ligand tuning ± drug co-treatment	Invasion; duct-like morphogenesis; mechanoresponsive signaling	Quantifies mechanical constraints; calibrates invasion/constraint terms in Model A	[43,44,46]
Stromal co-culture systems	PDO + CAFs ± endothelial cells in 3D matrices / microfluidics	Stromal-targeted agents; cytokine modulation; angiogenesis modifiers	Stromal activation; ECM remodeling; angiogenic structures	Captures tumor-stroma coupling; refines microenvironment priors for Model A	[38,48,49]
Immune-competent PDO models	PDO + T/NK/myeloid cells; ALI or organ-on-chip	ICB; co-stimulatory agonists; bispecifics; cytokines	Infiltration; cytotoxicity kinetics; activation/exhaustion markers	Links immune state to function; constrains immunotherapy modules	[13,50,51]
High-content imaging and AI phenotyping	Arrayed PDO cultures; microfabricated chips; longitudinal imaging	Dose matrices; combinations; schedule modulation; microenvironment cues	Single-organoid trajectories; morphodynamics; heterogeneity metrics	High-density phenotypes for atlas→ response mapping and Model A fitting	[54,55,69]
PDO multi-omics and longitudinal profiling	Serial sampling at baseline and multiple treatment time points	Time-resolved therapies; adaptation/holiday protocols	Transcriptomic/epigenomic/proteomic/metabolomic trajectories; persister states	Mechanisms of adaptation; parameterizes plasticity/time-varying resistance in the twin	[16,17,41]

PDO: patient-derived organoid; ICC: intrahepatic cholangiocarcinoma; ECC: extrahepatic cholangiocarcinoma; GBC: gallbladder carcinoma; HCC: hepatocellular carcinoma; ECM: extracellular matrix; CAF: cancer-associated fibroblast; SOC: standard of care; ALI: air-liquid interface; NK: natural killer; ICB: immune checkpoint blockade; AI: artificial intelligence.

### Quality control considerations for PDO-based and engineered models

The physical layer of a digital twin framework depends critically on the fidelity and reproducibility of PDOs and engineered tumor models. In practice, PDOs vary substantially in their ability to retain tumor-specific features, and experimental readouts can be influenced by culture conditions, batch effects, and biomaterial properties, as documented across multiple tumor types.^[59,94]^ As a result, functional data derived from these systems should not be treated as uniformly reliable by default, particularly when they are used to inform downstream modeling or clinical inference.

At a minimum, quality control (QC) should establish whether a given PDO captures key characteristics of the originating tumor. Histopathologic similarity and the expression of representative tumor markers provide a practical baseline for assessing tumor-PDO correspondence, while additional genomic or transcriptomic comparisons, when feasible, can further strengthen confidence in model fidelity.^[59]^

Reproducibility of functional readouts represents a second critical dimension of QC. Drug response measurements should demonstrate consistency across technical or biological replicates, and, where possible, across independent experimental batches, as variability at this stage has been shown to affect the interpretability of organoid-based screening results.^[94]^

For engineered tumor models, reporting of the ECM or scaffold context is essential for interpreting functional behavior. Even basic descriptors of matrix composition are informative, and quantitative parameters such as stiffness ranges can further contextualize observed phenotypes and treatment responses, given the established sensitivity of tumor cells to mechanical cues.^[16]^

In settings that incorporate co-culture or immune components, QC should document the presence and basic composition of these elements. While advanced immune or spatial readouts can add mechanistic insight, they are not a prerequisite for the use of PDO-based models in all translational scenarios.

Rather than serving as a binary pass-fail filter, QC information should inform how PDO-derived data are incorporated into the digital twin. Failure to meet minimal quality criteria should preclude data inclusion, whereas partial or variable quality may justify reduced confidence or weighting in model updating. To promote transparency and comparability across studies, we summarize a minimal QC checklist together with optional advanced assessments in Table 5.

**Table 5:** QC checklist for patient-derived organoids and engineered tumor models

QC category	Minimal QC	Optional advanced QC	Implications for data use
Tumor-PDO correspondence	Histopathology and marker expression	Genomic or transcriptomic concordance	Minimal QC required for inclusion; advanced concordance increases confidence
Functional reproducibility	Replicate consistency	Cross-batch reproducibility	Poor reproducibility triggers exclusion or reduced weighting
Biomaterial context	Matrix or scaffold type reported	Quantified stiffness or viscoelastic properties	Parameters contextualize functional readouts
Culture stability	Sustained growth and morphology	Longitudinal phenotypic stability	Unstable cultures unsuitable for longitudinal inference
Co-culture or immune context (if applicable)	Presence or absence documented	Functional or spatial immune readouts	Absence limits interpretation but does not preclude use

Minimal QC items define eligibility for primary use of PDO-derived data in Model A calibration. Datasets that fail minimal QC may still be informative for exploratory biology but should not be used for primary model fitting or high-confidence clinical interpretation. QC: quality control; POD: patient-derived organoid.

## The computational layer: architecting the digital twin

The “static atlases” described above (multi-omics, spatial omics, and pathology-based AI) primarily answer what the tumor and its microenvironment look like at a given time point, whereas “dynamic biomimetic models” (PDOs and their co-culture/organ-on-chip systems) are better suited to test what happens under a defined intervention. A digital twin connects these two views by integrating multi-scale data into a unified framework and by building patient-specific models that can be calibrated against experimental and clinical observations.^[95,96]^

To operationalize this vision, we outline a three-layer architecture that links data, computation, and clinical reality. The Data Layer organizes static and longitudinal information from digital pathology, spatial and single-cell omics, imaging, and routine clinical records into harmonized patient-level features. The Computational Layer hosts Model A and Model B within a closed-loop: Model A learns how atlas-derived baseline states relate to PDO phenotypes, and Model B uses these states to simulate patient-level trajectories under candidate interventions. The Physical Layer includes both PDO platforms and real patients; perturbation experiments and clinical treatments provide measured outcomes that feed back to refine data processing and recalibrate the coupled models over time, thereby turning the digital twin into a continuously learning cyber-physical system.^[17,95, 96, 97]^

### Model A: PDO twin trained on static atlases

Model A resides at the interface between the Data Layer and the Physical Layer. On the data side, it ingests multimodal atlases and curated clinical variables; on the physical side, it is anchored to baseline phenotypes from hepatobiliary PDOs and surgical specimens. Concretely, Model A learns a bidirectional mapping from high-dimensional static descriptors—such as tumor morphology, spatial ecology, and mutational or transcriptional programs—to a compact set of states that can be represented in simulations and instantiated in PDO experiments.^[98]^ This design allows Model A to translate heterogeneous patient data into experimental configurations that are feasible on organoid platforms while preserving constraints imposed by known hepatobiliary cancer biology. Accordingly, the descriptors that Model A must preserve are not identical across hepatobiliary entities: HCC mappings should retain vascular-associated and hypoxia-linked programs, whereas BTC mappings should retain stromal architecture and CAF-macrophage signaling states that dominate immune exclusion and drug penetration.^[31,33]^

Model A focuses on the organoid level and uses matched static-dynamic pairs to learn how a given baseline state translates into functional responses. From an implementation perspective, Model A can be specified by a well-defined set of inputs, outputs, and calibration targets. Typical inputs include atlas-derived baseline descriptors (*e.g*., bulk or spatial transcriptomic programs, histopathologic features, and selected clinical covariates) together with experimentally controllable context variables such as culture conditions or matrix type. The primary outputs are quantitative drug-response surfaces and phenotypic trajectories measured in matched PDOs, including viability, growth dynamics, and selected functional readouts.

Calibration of Model A is performed at the organoid level by aligning predicted response patterns with experimentally observed PDO measurements across replicates and perturbation conditions, rather than by fitting to patient outcomes directly. In practice, this calibration is constrained by limited sample sizes, incomplete omics coverage, and batch-to-batch variability, and therefore emphasizes reproducibility and internal consistency over exhaustive modality matching.

Inputs include multi-omics profiles, spatial features, and pathology-based readouts derived from the patient’s tumor, together with clinical covariates when available; outputs are drug responses and phenotypic trajectories measured in hepatobiliary PDOs and related co-culture or organ-on-chip systems.^[58,59,86]^ Trained on such aligned datasets, Model A summarizes organoid behavior in a way that remains anchored to genomics, histology, and clinical context, rather than to isolated experimental conditions.^[10,58,59,86]^

Because hepatobiliary PDOs are subject to selection, culture and measurement biases, building Model A depends on rigorous QC and standardized protocols. Rather than attempting to computationally “rescue” low-fidelity experiments, Model A is intended to learn treatment-response relationships and resistance trajectories that are established under reproducible conditions. In this sense, Model A provides a dynamic extension of static atlases: it translates baseline tumor states into experimentally verified response surfaces that will later inform and constrain patient-level modeling.^[59,86,99,100]^

Model A is attractive in principle, but its training data requirements are demanding. Robust learning depends on well-curated, patient-matched datasets that link baseline atlases (pathology ± spatial/multi-omics), controlled PDO context variables, and quantitative perturbation readouts—yet such paired datasets remain limited, fragmented, and often lack harmonized metadata and QC.^[13,77]^ Small sample sizes and laboratory-specific confounders (media, passage number, matrix type, imaging settings) increase the risk that models learn “site signatures” rather than transferable biology, undermining generalizability across centers.^[77]^ For this reason, Model A should prioritize reproducible experimental designs and transparent QC reporting, and use conservative learning strategies (*e.g*., regularization, external validation where possible) rather than maximizing model complexity in data-sparse settings.^[13,73]^

### Model B: Patient twin constrained by Model A

Model B focuses on longitudinal dynamics across the Computational and Physical Layers. Given an initial state inferred by Model A, Model B simulates how tumor burden, microenvironmental composition, and organ function evolve under specific drug regimens, radiotherapy schedules, or interventional procedures. In the *in vitro* setting, these trajectories can be partially observed through repeated PDO imaging, functional assays, and omics snapshots; in patients, they correspond to serial imaging, laboratory values, and clinical outcomes. Accordingly, Model B can be implemented as a dynamical model that combines a mechanistic structure with statistical or machine-learning components and can be calibrated using both organoid and clinical time series to maintain coherence between virtual and physical behavior.

Model B operates directly at the patient level. Its goal is to map multimodal baseline information—including clinical variables, imaging and pathology-derived features, as well as bulk or spatial multi-omics—onto clinical outcomes such as response, survival, and toxicity.^[99,101,102]^ In hepatobiliary cancers, many existing AI-based biomarkers derived from histopathology, radiology, and serum data can be regarded as early forms of Model B, although they typically do not include explicit constraints from PDO systems.^[6,103,104]^ Importantly, the clinical constraints that shape treatment feasibility and outcome differ between HCC and BTC, and Model B should encode these differences explicitly.

For HCC, hepatic reserve and portal-hypertension-related events often influence both treatment eligibility and survival independent of tumor burden; practical covariates include Child-Pugh or albumin-bilirubin (ALBI) grade, prior decompensation, and variceal/ascites history.^[50,105,106]^ For BTC, biliary obstruction and infectious complications can be the main determinants of whether systemic therapy can be delivered; bilirubin trends, drainage/stent status, and episodes of cholangitis therefore represent high-value covariates for longitudinal modeling and interpretation of outcomes.^[51,107]^

In real-world hepatobiliary cancer cohorts, calibration of Model B is often limited by sparse longitudinal follow-up, missing data modalities, and substantial treatment heterogeneity across patients. As a result, Model B is typically calibrated using partial trajectories and heterogeneous endpoints rather than dense, uniformly sampled time series. This practical constraint favors flexible modeling strategies that can accommodate irregular observation schedules and variable data availability, rather than assuming complete longitudinal profiles for every patient.

A defining feature of Model B in a digital-twin setting is its interaction with Model A. Instead of relying solely on observational cohorts, Model B can incorporate Model A-derived organoid response surfaces as prior information or constraints. When patient-level predictions deviate from PDO-based expectations, the discrepancy can highlight potential confounding or data shift, and motivate recalibration; when both levels agree, the resulting predictions carry stronger mechanistic support. Importantly, discordance between PDO-predicted responses and observed patient outcomes should not be interpreted as an automatic model failure. Such discrepancies may reflect unmodeled clinical confounders, differences in treatment exposure or adherence, or distribution shifts between *ex vivo* and *in vivo* contexts, rather than incorrect organoid phenotyping. In this setting, recalibration should be guided by uncertainty estimates and contextual evidence, rather than by enforcing agreement at every update step. In this way, Model A can reduce overfitting in Model B, while Model B extends organoid-derived insights to real-world clinical scenarios.^[59,95,108]^

Patient-level modeling is particularly vulnerable to overfitting and dataset shift because hepatobiliary cohorts are heterogeneous (treatment lines, follow-up schedules, missing modalities), and predicted probabilities can be poorly calibrated even when discrimination looks acceptable.^[109]^ For prediction models intended to support treatment decisions, calibration is as important as discrimination, because poorly calibrated probabilities can mislead even when ranking performance appears acceptable.^[110]^ The “black box” perception of many AI models remains a practical barrier to clinical trust, especially when models provide confident predictions without uncertainty estimates or a clear audit trail.^[18]^ Accordingly, digital-twin studies that include regression- or machine-learning-based prediction components should be reported in line with TRIPOD+AI and appraised with PROBAST+AI.^[109,111,112]^ Where feasible, internal-external or external validation across centers should be prioritized before clinical deployment, especially when the intended use spans heterogeneous treatment settings.^[113]^ These requirements also reinforce the value of using Model A-derived functional constraints as one safeguard against purely observational overfitting, while still treating discordance as a signal for confounding or shift rather than automatic model failure.^[18]^

### Closing the loop: In silico experimentation and feedback

Model A and Model B together form a feedback loop that links the Data, Physical, and Computational layers. Patient-derived data first define the baseline state of an individual tumor. Model A connects atlas-derived features with experimentally measurable responses in PDO systems. Model B then uses these states to simulate how the tumor may evolve under different treatment strategies. Based on these simulations, a small set of candidate strategies can be selected for experimental testing in the Physical Layer. These tests are usually performed through perturbation experiments in PDO platforms and, when possible, through clinical observation. The resulting measurements are then fed back into the system. They are used to update the Data Layer and recalibrate the models. In this way, the digital twin remains grounded in three sources of evidence: Atlas-derived data, organoid-based functional measurements, and real clinical outcomes.^[17,95, 96, 97]^ To illustrate how these components interact in practice, we outline a simplified single-patient workflow below.

Consider a patient with advanced intrahepatic CCA whose biopsy is obtained before first-line therapy. In routine workflows, the specimen is divided for different purposes. One part is used for standard histology and molecular profiling. Another fraction is used to establish PDOs. Sample triage procedures and pre-analytical information are recorded together with quality-control steps. Within the Data Layer, digital pathology features are combined with baseline imaging and laboratory measurements. These data are summarized into a compact representation of the tumor baseline state. In BTCs, this representation should capture several key constraints. These include stromal continuity and ductal-stromal boundaries, which often shape drug penetration. It should also include clinically important variables such as bilirubin trajectory, drainage or stent status, and recent episodes of cholangitis. These same features guide the design of the PDO model in the Physical Layer. For example, tumors with strong desmoplastic signatures may require stiffness-tunable matrices or fibroblast-enriched culture conditions, rather than standard epithelial organoid cultures.^[10]^

As the PDO reaches assay-ready expansion, a focused panel of candidate regimens is tested under standardized conditions to generate quantitative response readouts that can be compared across patients and timepoints, with uncertainty carried forward through replicate design and QC gates. In clinically annotated BTC series, PDO drug response has shown substantial concordance with observed patient outcomes, supporting its use as a practical “negative filter” to deprioritize likely-ineffective options when the decision space is large. Model A is then calibrated on the patient’s baseline atlas state together with PDO context variables and perturbation readouts, yielding an interpretable mapping between baseline features and functional sensitivities for that individual. Model B integrates this information with patient-level feasibility constraints and simulates longitudinal trajectories under candidate strategies; in HCC, analogous constraints would place greater weight on hepatic reserve and portal-hypertension-related variables, whereas in BTC, biliary obstruction and infection risk remain dominant.^[10]^ After treatment begins, incoming outcomes (radiographic response, bilirubin dynamics, adverse events, progression timing) are used for conservative recalibration and drift monitoring rather than unconstrained retraining, with updates documented transparently.^[114, 115, 116]^ Transparent reporting and risk-of-bias appraisal of the updated prediction workflow should follow emerging guidance for clinical prediction models that use regression or machine-learning methods.^[111,112]^

In practice, the loop depends on three key handoffs: A baseline state from the Data Layer, PDO perturbation readouts from the Physical Layer, and follow-up outcome data that can be used to update the Computational Layer.^[115,117]^

With these interfaces in place, once calibrated, the coupled system supports in silico exploration of treatment strategies. Virtual cohorts can be constructed to reflect the variation seen in hepatobiliary cancer patients as captured by atlases and clinical variables. For each virtual patient, Model B proposes candidate regimens, while Model A provides organoid-level expectations of response and resistance trajectories under the same regimens. These results can then be aggregated to compare expected benefit, risk, and common failure modes across strategies, helping prioritize clinical trial designs—especially for rare or heterogeneous subgroups.^[10,49,96,97]^

Rather than prescribing a fixed update cadence, the frequency and extent of model updating should depend on data availability, uncertainty estimates, and the clinical context in which new evidence is generated. In hepatobiliary cancers, such closed-loop digital twins do not replace prospective clinical evaluation, but they provide a structured way to integrate static atlases, dynamic PDO evidence, and longitudinal patient data. By iterating between prediction, experimental testing, and clinical observation, the framework can move toward models that are both mechanistically grounded and clinically usable (Table 6).

**Table 6:** Computational modules and closed-loop updating for hepatobiliary digital twins

Component	Typical Inputs	Calibration Target	Core modeling approaches	Primary outputs	Role in the Atlas-PDO-Twin loop	References
Model A: organoid-level twin	Latent states; PDO baseline omics; high-content phenotypes	Replicate-level PDO response patterns; limited paired atlas-PDO datasets and batch effects	Supervised/semi-supervised; hybrid mechanistic models	Predicted PDO responses; estimated growth/death/resistance parameters	Atlas state → *ex vivo* dynamics; constrains Model B	[10,15, 16, 17]
Model B: patient-level twin	Radiomics; clinical labs; treatment history; pathology/spatial-omics covariates; priors from Model A	Longitudinal clinical trajectories; sparse follow-up, missing modalities, treatment heterogeneity	State-space models; mechanistic simulators; deep time-series; RL frameworks	Trajectories of tumor burden, liver function, biomarkers, toxicity	Clinical evolution with PDO-grounded priors	[52,70,71]
Closed-loop calibration	PDO trajectories; clinical outcomes; prior parameter distributions	Predicted vs observed divergence over time; uncertainty propagation and data drift	Bayesian calibration; multi-level/multi-task learning	Parameter posteriors; refined atlas↔PDO↔ patient mappings	Aligns Model A/B as evidence accumulates	[11,12,68,69]
Virtual cohorts & *in silico* experimentation	Calibrated twins; population covariate distributions; treatment regimens	Benchmarked against observed population-level response, toxicity, and event-time distributions when available.	Virtual cohort simulation; causal inference; RL optimization	Response/toxicity distributions; optimized sequences/schedules	Regimen prioritization & trial design before prospective testing	[10,36,68,69]

PDO: patient-derived organoid.

## From in silico trials to patient benefit

“Static atlases” from pathology, multi-omics, and spatial profiling capture a patient’s tumor at baseline. What they cannot provide on their own is a forward-looking view of how the tumor will change once therapy starts. Digital twins may support more structured decision-making in hepatobiliary cancers. By connecting patient-level atlases with PDO-based functional data, they could screen out likely-to-fail regimens, identify testable vulnerabilities from TME features, and compare complex treatment combinations in virtual cohorts before prospective trials.^[10,86,96]^

Before discussing specific applications, it is useful to define a practical workflow for near-term implementation. In a realistic setting, a digital twin can be initialized from routine pathology, baseline imaging, and key laboratory data, while PDO establishment proceeds in parallel when feasible. If PDOs cannot be established or if the clinical time window is too short, the framework should default to an atlas-based prediction and use later PDO results, if available, to refine subsequent treatment decisions (Table 1).

### Individualized drug sensitivity: PDO-based decision support

The central role of PDOs is to translate patient-specific biology into reproducible and controllable functional readouts of treatment response. In practice, *ex vivo* drug sensitivity testing is most useful as a negative filter—it can flag regimens that are unlikely to help, rather than “guarantee” benefit from any single option.^[118, 119, 120]^ Evaluating PDO-guided clinical utility involves three criteria: technical feasibility (turnaround time and cost), clinical validity (correlation with outcomes), and clinical utility (improvement in patient management).^[118,119]^

Across multiple tumor types, prospective and clinically oriented studies suggest that PDO-based functional precision medicine can achieve high concordance between *ex vivo* drug response and clinical outcomes, particularly when pipelines and readouts are standardized in advance.^[118, 119, 120]^ In hepatobiliary cancers, emerging prospective datasets that link organoid experiments to treatment decisions and patient outcomes suggest that PDO-guided strategies may sharpen patient selection and improve treatment allocation.^[10,121]^ However, current cohorts remain modest and require further confirmation. In addition, PDO establishment and screening remain resource-intensive, and turnaround times are not always compatible with time-critical treatment decisions in routine care. A further barrier to clinical implementation is the lack of standardized protocols across centers for PDO establishment, culture conditions, drug testing, and response readout. These constraints should be made explicit when positioning PDO assays as decision-support tools rather than universal tests. Ultimately, whether PDO-guided decisions improve outcomes beyond standard-of-care algorithms should be tested in prospective studies, ideally randomized trials with prespecified endpoints.

In day-to-day practice, hepatobiliary cancers offer several settings where PDO+AI workflows are especially useful: Patients ineligible for standard clinical trials, individuals who have exhausted approved options, and cases with rare molecular profiles where guideline-directed therapy is uncertain. In these scenarios, the near-term goal is often to narrow the decision space by identifying clearly ineffective regimens, while integrating information from clinical variables, biomarkers, and imaging rather than replacing them.^[10,119]^ In practice, PDO-informed choices must be filtered through different clinical constraints: in HCC, liver function and portal-hypertension-related bleeding risk often limit feasible regimens, whereas in BTC, timely relief of biliary obstruction and infection control frequently determine whether systemic therapy can proceed.^[50,51]^

Conventional PDO drug screening often compresses response into a few discrete categories. Yet the underlying time- and morphology-resolved measurements contain richer signals, including growth kinetics, cell-death dynamics, and spatial response patterns. High-content analysis can convert these measurements into continuous sensitivity metrics and help detect heterogeneous subclones.^[81,122]^ It can also model combination effects, which improves the ability to discard non-beneficial regimens and to match patients to plausible sequences or combinations under real-world constraints. To make these criteria tangible, we summarize three representative hepatobiliary examples where patient-derived models were linked to treatment selection and/or clinical response alignment (Table 7).

**Table 7:** Three illustrative clinical translation vignettes linking patient-derived models to treatment response in hepatobiliary cancers

Case	Clinical context	Model	Intervention	Key clinical linkage	Translational takeaway	Reference
1	BTC	PDOs (± PDOX)	*Ex vivo* screening of SOC regimens	92.3% concordance with clinical response (12/13 evaluable); supported by PDOX	Supports regimen prioritization and exclusion of likely-ineffective options	[10]
2	HCC (anti-PD-L1)	Organoid-on-a-chip with MSC + PBMC (± CAF)	Functional response testing	More precise prediction potential for anti-PD-L1 response vs epithelial-only PDOs	TME-aware surrogates improve evaluation of microenvironment-dependent therapies	[13]
3	HCC-NED	PDOs (± PDOX)	*In vitro* drug sensitivity testing	Supported first-line etoposide + carboplatin; second-line entrectinib considered (organoid sensitivity + NTRK1); stopped at 2 weeks due to deterioration	Proof-of-principle in rare subtypes; highlights time-critical feasibility constraints	[100]

BTC: biliary tract cancer; CAF: cancer-associated fibroblast; HCC: hepatocellular carcinoma; HCC-NED: HCC with neuroendocrine differentiation; MSC: mesenchymal stromal cell; PBMC: peripheral blood mononuclear cell; PD-L1: programmed death-ligand 1; PDO: patient-derived organoid; PDOX: PDO-derived xenograft; SOC: standard of care; TME: tumor microenvironment.

### Mechanistic insight and target discovery

High-dimensional static atlases in hepatobiliary cancers repeatedly reveal feature axes linked to immune infiltration, stromal remodeling, metabolic reprogramming, and malignant-cell differentiation state. These axes are associated with clinical outcomes and treatment response, and often emerge consistently across tissue- and molecular-level modalities.^[30,70,123,124]^ For example, single-cell dissection of AFP-positive HCC delineates an immunosuppressive niche dominated by SPP 1^+^ TAMs and exhausted T cells, and demonstrates that blocking the SPP1-CD44 axis can reinvigorate T-cell function and reduce tumor burden in preclinical models.^[125]^ Likewise, a comprehensive single-cell and spatial analysis of GBC shows that epithelial OLFM4 not only marks disease progression but also drives PD-L1 upregulation and T-cell dysfunction through MAPK-AP1 signaling, nominating OLFM4 as a candidate biomarker and therapeutic target in GBC.^[126]^ A central task of digital twins is to turn these descriptive axes into testable hypotheses about vulnerabilities within specific cellular compartments and ecological niches.

To do so, candidate targets or pathways identified from spatial omics and single-cell analyses can be mapped onto PDOs and co-culture systems that better capture TME composition and organization. Ecological PDO models that incorporate immune cells, fibroblasts, and endothelial components allow targeted perturbations to be tested in a controlled but still context-rich setting.^[127, 128, 129, 130]^ Examples include checkpoint signaling, stromal growth factors, and metabolic dependencies. Within a digital-twin loop, organoid perturbation data can then refine atlas-derived candidates and feed back into patient-level models that estimate which subpopulations are most likely to benefit. In hepatobiliary tumors, matched organoid systems have also been used to connect neoantigen prediction with functional T-cell assays, illustrating how organoids can narrow immunotherapy hypotheses at the individual-patient level.^[131]^

Once static atlases and ecological PDO models converge on a set of candidate pathways or molecules, genetic screening can further narrow the search space and strengthen causal inference. Barcode-assisted CRISPR/CRISPRi/CRISPRa screens in three-dimensional organoid platforms have demonstrated that genetic effects on growth and drug response can be assessed at scale while preserving tissue architecture and cell-cell interactions.^[92,93]^ Combined with multi-omic readouts and atlas-based priors, these pooled perturbation frameworks help prioritize targets that not only produce robust phenotypes in PDOs but also align with TME-defined vulnerabilities in patients, reinforcing the closed-loop architecture between atlas, model, and functional validation.^[70,86,123]^

### Optimizing new drugs and treatment strategies

Systemic therapy for hepatobiliary cancers is marked by modest single-agent efficacy, rapidly expanding options, and increasingly complex combinations spanning targeted therapy, anti-angiogenic agents, and immunotherapies. The number of plausible regimens and sequences greatly exceeds what can be reasonably tested in conventional trials. Digital twins that integrate static atlases, PDO-based functional readouts, and clinical variables offer a structured way to explore this combinatorial space and to prioritize strategies for formal evaluation.^[10,96,132]^

For combination and sequential regimens, digital twins shift the emphasis from “which drugs to choose” to “how to administer them over time”. Digital twins can simulate treatment schedules, dose intensity, and switching criteria in silico. When these simulations are constrained by pharmacokinetics, toxicity, and real-world feasibility, they can identify regimen regions that are unlikely to be effective or tolerable, reducing the experimental burden before prospective testing.^[56,122,133]^

Virtual trials and synthetic patients provide a complementary approach. Here, individualized twins calibrated on real-world datasets or trial data are assembled into in silico cohorts that reproduce key dimensions of clinical heterogeneity. These virtual trials do not replace prospective studies, but they can inform dose selection, biomarker-enriched eligibility criteria, and arm prioritization by estimating expected benefit within predefined subgroups.^[95,96,134]^ More broadly, recent scoping reviews suggest that digital-twin use in healthcare is expanding rapidly, but robust prospective deployment for clinical decision-making remains limited.^[135,136]^

To translate these tools into actionable plans, clear data flows, and transparent model reporting are needed. Governance is also required to define how in silico evidence should be weighed alongside clinical, radiologic, and pathologic information. Prospective, pre-registered evaluations, shared benchmarks, and interoperable pipelines will be critical to avoid opaque recommendations and preserve clinician trust.^[96,108,137]^ Ultimately, the contribution of digital twins to new drug and strategy development in hepatobiliary cancers will depend on how effectively computational modeling, organoid experimentation, and clinical decision-making are connected in a coherent and interpretable loop.^[96]^

Overall, the translational value of digital twins in hepatobiliary cancers depends on a loop that is not only closed, but testable and iterative. Computational models can deprioritize combinations, sequences, or targets that are unlikely to succeed and elevate those consistent with atlas-derived vulnerabilities. PDOs, co-culture systems, and organ-on-chip platforms then provide functional validations that help eliminate ineffective regimens and sharpen mechanistic interpretation. Clinical data—from real-world evidence to early-phase trials—should then be used to recalibrate model parameters, improve prediction performance, and define the subgroups most likely to benefit. Together, this closed-loop framework supports the rational development of new drugs and treatment strategies, and provides a practical path for integrating digital twins into precision oncology workflows.^[96]^

## Summary and outlook

At present, digital twins in hepatobiliary oncology are better regarded as an emerging translational strategy than as a mature clinical tool. The most realistic near-term application is not a fully comprehensive twin for every patient, but a more focused framework that combines selected baseline data with functional readouts when feasible.^[30,70,86]^ In this review, we discuss digital twins as a framework that connects data, experimental models, and longitudinal clinical information. By integrating multi-omics, bioengineering, and AI, this framework links data, experimental models, and longitudinal clinical information to support dynamic modeling of treatment response and resistance.^[12,95]^

A practical way to organize this framework is the Atlas-PDO-Twin loop, which links baseline data, functional testing, and follow-up observations. In this recursive process, static data initializes the model, PDOs validate mechanistic hypotheses, and the digital twin synthesizes these inputs to guide clinical strategy.^[10,12,95]^ This approach is particularly critical for hepatobiliary cancers—including HCC, CCA, and GBC—where profound heterogeneity, desmoplasia, and immune evasion render generic treatment guidelines insufficient.^[19,70]^ Moving beyond descriptive studies, the field is now advancing toward predictive systems where heterogeneity is no longer a barrier to treatment, but a computable dimension of precision care.^[12,95]^

A practical cancer digital twin will only be as reliable as the data and biological surrogates that anchor it. In multi-center settings, seemingly small pre-analytical differences—ischemia time, fixation, and library preparation—together with batch effects and missing metadata can measurably shift multimodal readouts and complicate cross-study comparisons.^[138,139]^ PDOs add a functional layer, but most systems still lack perfusable vasculature and neural inputs and incompletely reproduce biliary flow, endocrine cues, and long-range immune-stromal dynamics, which can bias drug penetration and therapy-induced remodeling.^[140]^ Another practical issue is state drift: twins are often initialized from a single biopsy, while clinical trajectories unfold over months to years; longitudinal updating with serial imaging, circulating biomarkers, and repeat sampling where feasible is therefore essential if the twin is to behave as a state-estimation system rather than a one-time predictor.^[13]^ Finally, scalability will hinge on whether organoid-enabled workflows can be delivered within a clinically relevant window, with acceptable success rates and costs; tiered pipelines and early assessment of resource burden may help move from bespoke demonstrations to sustainable deployment.^[73,77]^

Despite this momentum, significant bottlenecks impede the translation of digital twins from proof-of-concept to standard clinical practice. At the data level, the prospective acquisition of harmonized, multi-modal datasets remains a formidable challenge, as inconsistencies in sampling and processing across cohorts complicate external validation and model generalization.^[18,19]^ Simultaneously, in terms of model fidelity, balancing mechanistic interpretability with computational performance remains an open question, particularly when integrating incomplete data modalities.^[12,95]^ Finally, regarding clinical integration, the high cost, extended turnaround time, and complexity of high-fidelity PDO workflows pose substantial logistical barriers.^[10,86]^ Addressing these hurdles will require not only technological refinement but also the establishment of robust governance frameworks to manage data security, interoperability, and the ethical use of synthetic patient data.^[12,96]^

Beyond these logistical hurdles, a more profound scientific challenge lies in synchronizing the disparate time scales of the twin’s components: cellular signaling events occur in seconds, organoid drug responses evolve over weeks, while clinical tumor progression is monitored over months or years. Current models often ignore this temporal mismatch. Future architectures must therefore develop “temporal alignment algorithms” capable of extrapolating acute, short-term organoid responses to long-term patient outcomes, rather than assuming a simple linear correlation between the two.

Looking ahead, the realization of clinical-grade digital twins will require concerted efforts in four key directions. First, the community must establish unified data representations that bridge tissue, organoid, and clinical phenotypes to ensure model interoperability.^[19,30]^ In practice, this will likely depend on scalable multimodal learning—such as foundation-style encoders trained across pathology, radiology, and omics—to produce transferable embeddings that remain usable even when certain modalities are missing. Second, next-generation “ecological” PDOs—incorporating autologous immune and stromal components—are essential to minimize the fidelity gap between *ex vivo* models and *in vivo* biology.^[10,70,86]^ Third, research must pivot from retrospective correlations to prospective, interventional trials where digital twins actively guide treatment selection and are iteratively refined by real-world outcomes.^[12,95]^ Here, hybrid strategies that couple mechanistic models with data-driven learning can improve interpretability and support counterfactual testing, rather than treating the twin as a black box. Finally, multicenter consortia are indispensable for establishing the standardization and governance required for regulatory acceptance, and privacy-preserving approaches (*e.g*., federated learning with secure infrastructures) will be important for scaling across hospitals without centralizing sensitive patient data.^[12]^

In conclusion, the integration of high-resolution atlases, functional PDO systems, and emerging digital-twin frameworks is reshaping how hepatobiliary cancers are characterized and managed. By unifying tissue context, functional readouts, and clinically relevant simulations, these approaches offer a path toward predictive, continuously learning models that support more proactive and individualized treatment decisions. Achieving this vision will require coordinated advances in data standardization, model validation, and clinical implementation. Current trajectories suggest that, as these systems mature, digital twins may transform tumor heterogeneity from a barrier into an actionable dimension of precision oncology.
